# Anthropometric surrogates to identify low birth weight Nepalese newborns: a hospital-based study

**DOI:** 10.1186/1471-2431-8-16

**Published:** 2008-04-25

**Authors:** Chandrashekhar T Sreeramareddy, Neena Chuni, Rajkumar Patil, Dela Singh, Brishna Shakya

**Affiliations:** 1 Department of Community Medicine, Manipal Teaching Hospital, Manipal College of Medical Sciences, Pokhara, Nepal; 2 Department of Obstetrics and Gynecology, Manipal Teaching Hospital, Manipal College of Medical Sciences, Pokhara, Nepal; 3 Department of Pediatrics, Manipal Teaching Hospital, Manipal College of Medical Sciences, Pokhara, Nepal; 4 Department of Obstetrics and Gynecology, Western Regional Hospital, Pokhara, Nepal

## Abstract

**Background:**

In Nepal, more than 90% of the deliveries take place at home where birth weight is often not recorded. In developing countries, low birth weight (LBW, <2500 grams) accounts for 60–80% of neonatal deaths. Early identification and referral of LBW babies for extra essential newborn care is vital in preventing neonatal deaths. Studies carried out in different populations have suggested that the use of newborn anthropometric surrogates of birth weight may be a simple and reliable method to identify LBW babies in a home setting. However, a reliable anthropometric surrogate to identify LBW babies and its cut-off point is not known for Nepalese newborns.

**Methods:**

A cross-sectional study was carried out in Western Regional Hospital, Pokhara between April and June, 2006. All consecutive full-term, singleton, live born babies were included. To ensure reliability and avoid inter-observer bias one of the investigators weighed all the newborns and carried out anthropometric measurements within 24 hours after birth. Circumferences of head, chest, mid-upper arm, thigh and calf were measured according to standard techniques. Non-parametric receiver operating characteristic (ROC) curve analyses were carried out using bootstrap to calculate 95% confidence intervals of areas under the curve (AUC). The cut-points with lowest total misclassification rate were chosen to identify LBW babies.

**Results:**

Out of 400 newborns studied, 204 (51%) were males and 196 (49%) were females. The mean birth weight was 3029 ± 438 grams and 34 (8.5%) newborns were LBW. By ROC-AUC analyses, head circumference (AUC = 0.89, 95% CI 0.85 to 0.93) and chest circumference (AUC = 0.86, 95% CI 0.80 to 0.91) were identified as the optimal surrogate indicators of LBW babies. The optimal cut-points for head circumference and chest circumference to identify LBW newborns were ≥ 33.5 cm and ≥ 30.8 cm respectively.

**Conclusion:**

Head and chest circumferences were the best anthropometric surrogates of LBW among Nepalese newborns. Further studies are needed in the field to cross-validate our results.

## Background

Of the approximately four million global neonatal deaths that occur annually, 98% occur in developing countries, where most newborns die at home while they are being cared by mothers, relatives, and traditional birth attendants [1]. About 38% of total under-five mortality occurs during the first 28 days of life and nearly three quarters of these deaths occur during the first week of life [2]. Globally, about one-sixth of all newborns are low birth weight (LBW, <2500 grams), which is single most important underlying risk factor for neonatal deaths [1,3]. Only about half of the newborns are weighed at birth and for a smaller proportion of them gestational age is known [4]. An estimated 18 million babies are born with LBW and half of them are born in south Asia [5]. Although these LBW babies account for 14% of the children born, they account for 60–80% of neonatal deaths [6]. Moreover, LBW babies who survive the critical neonatal period may suffer impaired physical and mental growth. Therefore, an early identification and prompt referral of LBW newborns is vital in preventing neonatal deaths. Available evidence from resource-poor settings shows that extra essential newborn care for LBW babies can reduce the number of neonatal deaths by 20–40% [7]. Research has also shown that this extra essential newborn care may be delivered by health workers or family members if they are suitably trained [8]. In resource-poor settings, a large proportion of deliveries take place at home and birth-weight is most often not recorded. Therefore, there is a need to develop simple, inexpensive and practical methods to identify LBW newborns soon after birth [9]. One such method may be the use of anthropometric surrogates to identify LBW babies.

Several researchers have attempted to identify suitable anthropometric surrogates which are simple and reliable to identify LBW babies. Recent hospital-based studies from India, Bangladesh and other developing countries have suggested different anthropometric surrogates to identify LBW babies and have also recommended various cut-off values for identification of LBW babies [10-19]. Available evidence suggests that there is a lack of consensus about most reliable anthropometric surrogate and a fixed cut-off point. A multi-center study carried out by the World Health Organisation (WHO) reported that validity of mid-upper arm circumference (MUAC) and chest circumference (CHC) and cut-off points for identifying LBW babies varied across the nations and ethnic groups. Nepal was not included in the WHO multi-center study [20]. Therefore, there is a need to identify a suitable anthropometric surrogate and define its cut-off point for different populations.

Nepal is a Himalayan kingdom located between China and India. In 2004, infant mortality rate was 64 per 1000 live births and neonatal mortality rate was 39 per 1000 live births [21]. In rural areas of Nepal, proportion of institutional deliveries is as low as four percent [22,23]. Even in urban areas like Kathmandu [24], and Pokhara [25] a significant proportion of women give birth at home. Skilled attendance at birth is very low and most often birth weight is not recorded [22,23,25]. It is reported that in many developing countries birth weight is often not recorded due to lack of weighing scales or logistic problems in using available scales in home setting [20]. Trained traditional birth attendants (TBAs) and maternal child health (MCH) workers who are considered as key birth attendants do not posses a weighing scale in their delivery kits [21]. Lack of recording birth weight may be responsible for high infant mortality rate in Nepal. Therefore, it is important to identify the LBW babies who are often born at home. Most suitable and reliable anthropometric surrogate to identify LBW Nepalese newborns and its cut-off point to identify LBW newborns is not known. Therefore, we carried out this study with following objectives

1) to identify a suitable anthropometric surrogate to identify LBW babies and

2) to determine its cut-off value to identify LBW babies.

## Methods

A cross-sectional study was carried out in Western Regional Hospital, Pokhara, Nepal, during the time period April-June, 2006. Western Regional hospital is a teaching hospital affiliated to Manipal College of Medical Sciences (MCOMS). The institutional research ethics committee of MCOMS approved this study. All the consecutive full-term, singleton, live born babies during the study period were included for the study. The newborns with congenital anomalies/dysmorphic features, multiple births and gestational age of less than 37 completed weeks (pre term babies) were excluded. To ensure reliability and avoid inter-observer bias, one of the investigators (Brishna Shakya) weighed all the newborns and carried out all anthropometric measurements within 24 hours after birth. The investigator obtained informed consent from the mothers to examine their newborn. Gestational age of the newborns was obtained from the medical case file. Circumferences of head, chest, mid-upper arm, thigh and calf were measured to the nearest 0.1 cm using a non-elastic, flexible, fiber glass measuring tape according standard techniques described by Jelliffe [26].

All the newborns were weighed naked on a spring type of weighing scale to the nearest 50 grams. The weighing machine was checked daily by known standard weight before weighing. Mid-upper arm circumference (MUAC) was measured at the midpoint between the tip of acromion process and olecranon process of the left upper arm. Head circumference (HC) was measured between glabella anteriorly and along the most prominent point posteriorly. Chest circumference (CHC) was measured at the level of nipple at the end phase of expiration. Thigh circumference (TC) was measured at the lowest furrow of gluteal region. Calf circumference (CC) was measured at the most prominent point in a semi-flexed position of the leg.

### Statistical analysis

Continuous variables are reported as mean and standard deviation while categorical variables are given as the number or the percentage of subjects with the characteristic of interest. Between-gender comparisons of continuous variables were performed using student's unpaired *t*-test. Pearson's product-moment correlation coefficient was used to assess the association between anthropometric measurements. Receiver operating characteristic (ROC) curves were used to evaluate the accuracy of different anthropometric measurements to predict LBW coded as dichotomous (1 = yes; 0 = no). The area under the ROC curve (AUC) was calculated using the non-parametric method of De Long. Internal cross-validation was performed by calculating 95% confidence intervals (95%CI) of ROC AUCs on 1000 bootstrapped samples of 400 subjects. Sensitivity, specificity and positive likelihood ratios were calculated at all cut-points for any anthropometric measurement. We choose as "optimum" the cut-point with the highest [(sensitivity + specificity)/2] ratio, i.e. the lowest total misclassification error rate. This criterion was chosen to allow comparison with previous studies available in the literature. A p-value < 0.05 was considered statistically significant. All statistical tests are two-tailed. Statistical analysis was performed using SPSS 10 and STATA 10.0.

## Results

During the study period, a total of 613 deliveries took place in the hospital. Two hundred and thirteen deliveries were excluded from the study for the following reasons: 1) age > 24 hours (n = 9), 2) preterm birth (n = 184), 3) multiple births (n = 12), 4) still births (n = 6) and, 5) congenital anomalies or dysmorphic features (n = 2). A total of 400 newborns (51% males and 49% females) were therefore studied. Their median age was eight hours (inter-quartile range 4 to 12 hours) and their anthropometric measurements are given in Table 1. All anthropometric measurements were similar in males and females. Expectedly, the anthropometric measurements were highly correlated (Table 2), with the best correlation coefficient observed for the weight-head circumference association.

**Table 1:** Descriptive statistics of birth weight and anthropometric measurements

	Male	Female	Total
	
Parameter	Mean (SD)	Mean (SD)	Mean (SD)
Birth weight (grams)	3031 (436)	3026 (441)	3029 (438)
HC (centimeters)	34.7 (1.4)	34.4 (1.5)	34.6 (1.5)
CHC (centimeters)	32.8 (1.8)	32.8 (2)	32.8 (1.9)
MUAC (centimeters)	11.4 (1.1)	11.4 (1.1)	11.4 (1.1)
TC(centimeters)	14.5 (1.3)	14.6 (1.4)	14.6 (1.3)
CC (centimeters)	11.7 (1.2)	11.6 (1.2)	11.7 (1.2)

**Table 2:** Zero-order correlation matrix and Pearson's correlation coefficients

	Birth weight	HC	CHC	MUAC	TC	CC
Birth weight	1.000	0.744	0.701	0.631	0.683	0.553
HC	-	1.000	0.653	0.609	0.667	0.525
CHC	-	-	1.000	0.663	0.644	0.634
MUAC	-	-	-	1.000	0.696	0.736
TC	-	-	-	-	1.000	0.672
CC	-	-	-	-	-	1.000

All the correlations were significant at the 0.01 level.

**Figure 1:**
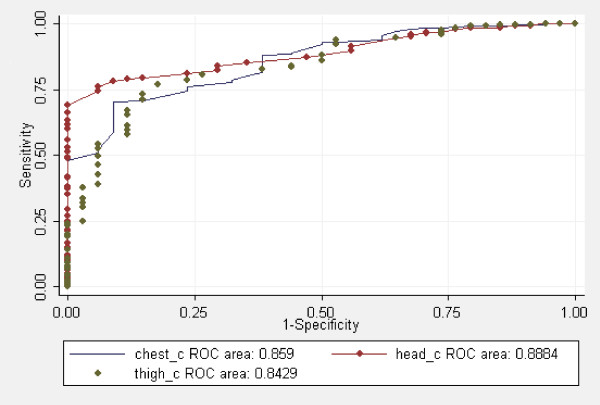
**Comparison of ROC curves for HC, CHC and TC to choose optimal surrogate for Birth weight**. All the comparisons were statistically significant (p < 0.001)

Thirty four of the 400 (8.5%) newborns were LBW. The best discrimination of LBW, as detected by ROC-AUC, was obtained by head circumference (AUC = 0.89, 95% CI 0.85 to 0.93) followed by chest circumference (AUC = 0.86, 95% CI 0.80 to 0.91), calf circumference (AUC = 0.85, 95% CI 0.76 to 0.93), thigh circumference (AUC = 0.84, 95% CI 0.78 to 0.91) and arm circumference (AUC = 0.83, 95% CI 0.77 to 0.89) (Figure 1). Although there was not enough power for a formal comparison of ROC-AUCs because of the low number of positive outcomes, we considered head circumference as the best surrogate measure of LBW. Table 3 gives sensitivity, specificity and positive predictive values for the cut-points of head, chest and thigh circumference associated with the lowest total misclassification rate.

**Table 3:** Validity of head, chest and thigh circumferences to identify LBW newborns

Measurement	Critical limit (Centimeters)	Sensitivity	Specificity	Positive Predictive Value
HC	33.5	81.15%	76.47%	97.4
CHC	30.8	87.98%	61.76%	96.1
TC	13.7	78.69%	76.47%	97.9

## Discussion

In our study there were no significant differences in birth weight and anthropometric measurements between male and female newborns. Therefore we analyzed the combined data for both sexes. Mean birth weight in our study was relatively higher than the previous studies from India and Bangladesh. A WHO multicenter study reported that the average birth weight was 2630, 2780 and 3840 for newborns in India, Nepal and Sri Lanka respectively [27]. Higher mean birth weight may be because only the full term singleton live births were included in our study. Previous studies did not specify such criteria [11,14-20]. Birth weights of the newborns born before completion of 37 weeks of gestation (full term) may also have been included in the studies cited above. The proportion of LBW was low (8.7%) in our study which is in contrast to similar studies reported earlier where the proportion of LBW varied from 10% to 46% [11,15-19]. However, a majority of these studies may have included preterm newborns also. This is because the authors did not mention such criteria. Reliable population-based data on proportion of LBW in Nepal is not available. The Nepal Demographic and health survey reported that up to 40% of the newborns in rural areas are LBW [28].

Many researchers have attempted to identify a suitable anthropometric surrogate to identify LBW babies which is reliable, simple, and logistically feasible in field conditions. Some studies have recommended that CHC, MUAC and HC may be used as anthropometric surrogates to identify LBW babies [9,12-16,18-20]. Some other studies have recommended CC and TC as suitable anthropometric surrogates to identify LBW babies [10,11,17]. Therefore we considered all these anthropometric measurements. A recent report from Sarlahi district reported that chest circumference was superior to foot length as an anthropometric surrogate to identify LBW babies [30]. However this study did not compare other anthropometric surrogates with birth weight to select an optimal surrogate. None of the studies reported earlier have compared foot length with birth weight [10-20]. In our study HC was identified as a suitable surrogate to identify LBW babies. Some studies have identified MUAC as a suitable anthropometric surrogate for birth weight [14-16,19].

It is argued that measurement of HC may not be accurate due to moulding of head during birth especially during prolonged and obstructed labor [15]. Previous studies have suggested that CHC was a better surrogate for birth weight [9,20,30]. A community-based study from Sarlahi has also recommended CHC to be superior to foot length [30]. Therefore, we also consider that CHC may be a better anthropometric surrogate for identifying LBW newborns in Nepal's context. Health workers may be trained to identify LBW babies by measuring CHC. It has been suggested that measuring CHC is simpler because identification of nipple line is relatively easier than other measurements [20,30]. Therefore, CHC may be operationally more feasible. CHC may have a drawback in Nepal's context with reference to maintenance warm chain for the newborn especially during the months of winter. This is because it is necessary to remove waddling clothes to measure CHC. A WHO collaborative study has recommended that CHC of 29 centimeters and 30 centimeters may identify 'highly at risk' and 'at risk' newborns respectively [20]. In our study maximum sensitivity and specificity for CHC was at CHC of 30.8 centimeters. The higher mean birth weight of newborns may be the reason for a slightly higher cut-off point obtained in our study. We considered only full-term deliveries, which was unlike earlier studies [9,12-19]. Although some studies have shown MUAC, CC and TC to be superior for identification of LBW newborns, chest circumference has generally shown to be a better surrogate measure for identification of LBW newborns [9,20,30]. The cut-off points we obtained by ROC curve analysis are relatively higher than those suggested by previous studies [20,30]. Further studies are necessary to define a more precise cut-off point for Nepalese newborns. The cut-offs suggested in our study were not accurate. Similar results were observed in the WHO multicenter study. The sensitivity and predictive accuracies of CHC for identifying LBW newborns widely varied across different study samples for different cut-offs i.e. 28, 29, 30 centimeters [20]. The decision on choice of a cut-off point may depend on the resources available to manage the LBW (high risk) newborns in the community. It is suggested that the family members or health workers who usually attend the deliveries at home may be given cut-off rules with lesser precision (0.5 centimeter) [30].

A study from rural Sarlahi district, Nepal reported that a low cost, colour coded, hand-held spring scale can accurately categorize birth weight. The study has also reported that the accuracy of such device exceeded that of anthropometric surrogates to identify LBW babies [9]. Further studies may be required to test the validity of such a device and operational feasibility of its use in home setting. In accordance with previous researchers we also recommend that a color coded, non-elastic, flexible measuring tape may be used in Nepal [14,15,20,30]. A three color coded tape similar to Shakir's tape which is used to identify the children with undernutrition may be suggested to overcome the problems of illiteracy. We suggest that clean home delivery kits which are currently manufactured and promoted in Nepal may contain a color coded measuring tape. Recently a financial incentive scheme has been implemented in Nepal to improve the uptake of institutional deliveries. Under this scheme the skilled birth attendant during delivery receives a financial incentive [21]. So we anticipate that the presence of a skilled attendant and use of CHDK may improve during home delivery. Therefore it is necessary to define the optimal cut-offs and validate the use of such device by lesser trained health workers or family members in home setting.

To our knowledge this is the first study to identify the anthropometric surrogates to identify LBW babies for Nepalese newborns. Such study was necessary since the WHO multi center study did not include Nepal among its study sites [20]. Moreover, the predominance of tibetoburmese ethnic group in Nepalese population [29] warranted such a study. The mean birth weight (3029 ± 438 grams) of full term newborns in our study was similar to that noted among the two samples of newborns examined in China during the WHO multicenter study on surrogate indicators to identify LBW babies [20]. There is a need for further studies to validate our results and to define optimum cut-offs for the appropriate surrogates to identify LBW newborns.

Our study has some limitations. The proportion of LBW newborns was rather low in our study since only full-term singleton births were included. Therefore, the positive predictive values were lower in our study. The potential bias was eliminated by the same investigator taking all measurements within 24 hours after birth and daily checking of the weighing scale with a known standard. However, there could have been some intra-observer bias in measurements. Since this study was carried out on a sample of newborns babies in a hospital generalizability of the findings to the community may be limited. Poor precision (nearest 50 grams) of spring type of weighing scale used in our study was another limitation. Further studies on a community-based sample of newborns using a more precise weighing scale and by measuring all the suggested anthropometric surrogates may be necessary. Moreover, the suggested surrogates to identify LBW newborn should be validated in the field setting. This is because the surrogate measurements are intended to be used by health workers in the field setting. Community-based studies may have to overcome the time delay involved in taking measurements. It is well known that a newborn looses weight during the first 72 hours after birth [31,32]. We included only full-term newborns since maximum overall growth of the fetus can be assessed among full-term newborns. Preterm and multiple pregnancies may be either appropriate for gestational age or small for date. Therefore, only full term newborns were selected to avoid such confusion during interpretation of our results. However, our results may be applied in the community setting where gestational age is often difficult to ascertain. The newborns who are preterm and LBW are also at more risk of morbidity and mortality. Therefore, identifying any LBW newborns whether small-for-date or preterm or both is important for reduction of neonatal mortality.

## Conclusion

The results of our study suggest that chest circumference may be an optimum anthropometric surrogate to identify LBW Nepalese newborns. Further studies are required to validate our results in the field setting and define an optimal cut-off value. A color coded, measuring tape may be suggested for use by health workers or family members to identify LBW newborns in home setting.

## Competing interests

The authors declare that they have no competing interests.

## Authors' contributions

CTS contributed to the design and protocol of the study, assisted in the data collection, was the primary researcher and drafted the manuscript for publication. NC contributed to the design and protocol of the study, assisted in the data collection, was the primary researcher and drafted the manuscript for publication. RP helped in conception of the study, designed data entry and analysis. DS provided assistance during the data collection, analyses and interpretation, criticized the earlier drafts of the manuscript for its intellectual content. BS carried out all the anthropometric measurements and assisted in manuscript preparation. All authors read and approved the final manuscript for submission for Publication.

## Pre-publication history

The pre-publication history for this paper can be accessed here:



## References

[B1] World Health Organization (1996). Perinatal mortality: a listing of available information. FRH/MSM967 WHO, Geneva.

[B2] Lawn JE, Cousens S, Zupan J, Lancet Neonatal Survival Steering Team (2005). 4 million neonatal deaths: when? Where? Why?. Lancet.

[B3] Save the Children Federation (2001). World Health Organisation 2001 estimates. Saving newborn lives, state of the world's children Washington, DC.

[B4] Blanc AK, Wardlaw T (2005). Monitoring low birth weight: an evaluation of international estimates and an updated estimation procedure. Bull World Health Organ.

[B5] United Nations Children's Fund (UNICEF) (2005). The state of the world's children New York.

[B6] Bang A, Reddy MH, Deshmukh MD (2002). Child mortality in Maharashtra. Economic Political Weekly.

[B7] Darmstadt GL, Bhutta ZA, Cousens S, Adam T, Walker N, de Bernis L, Lancet Neonatal Survival Steering Team (2005). Evidence-based, cost-effective interventions: how many newborn babies can we save?. Lancet.

[B8] Bang AT, Bang RA, Baitule SB, Reddy MH, Deshmukh MD (1999). Effect of home-based neonatal care and management of sepsis on neonatal mortality: field trial in rural India. Lancet.

[B9] Mullany LC, Darmstadt GL, Coffey P, Khatry SK, LeClerq SC, Tielsch JM (2006). A low cost, colour coded, hand held spring scale accurately categorises birth weight in low resource settings. Arch Dis Child.

[B10] Naik DB, Kulkarni AP, Aswar NR (2003). Birth weight and anthropometry of newborns. Indian J Pediatr.

[B11] Samal GC, Swain AK (2001). Calf circumference as an alternative to birth weight to predict low birth weight babies. Indian Pediatr.

[B12] Gupta V, Hatwal SK, Mathur S, Tripathi VN, Sharma SN, Saxena SC, Khadwal A (1996). Calf circumference as a predictor of low birth weight babies. Indian Pediatr.

[B13] Verma SS, Ghadiok AK, Kishore N, Singh OP (1996). Head and chest circumferences as predictors of low birth weight in Indian babies. J Trop Pediatr.

[B14] Das JC, Afroze A, Khanam ST, Paul N (2005). Mid-arm circumference: an alternative measure for screening low birth weight babies. Bangladesh Med Res Counc Bull.

[B15] Dhar B, Mowlah G, Nahar S, Islam N (2002). Birth-weight status of newborns and its relationship with other anthropometric parameters in a public maternity hospital in Dhaka, Bangladesh. J Health Popul Nutr.

[B16] Ahmed FU, Karim E, Bhuiyan SN (2000). Mid-arm circumference at birth as predictor of low birth weight and neonatal mortality. J Biosoc Sci.

[B17] Arisoy AE, Sarman G (1995). Chest and mid-arm circumferences: identification of low birth weight newborns in Turkey. J Trop Pediatr.

[B18] Ezeaka VC, Egri-Okwaji MT, Renner JK, Grange AO (2003). Anthropometric measurements in the detection of low birth weight infants in Lagos. Niger Postgrad Med J.

[B19] Hossain MM, Habib M, DuPont HL (1994). Association between birth weight and birth arm circumference of neonates in rural Egypt. Indian J Pediatr.

[B20] WHO (1993). Use of a simple anthropometric measurement to predict birth weight. WHO Collaborative Study of Birth Weight Surrogates. Bull World Health Organ.

[B21] Ministry of Health, His Majesty Government (2004). Annual Health Report-2004.

[B22] Ministry of Health, Family Health Division (1998). Maternal mortality and morbidity study Kathmandu.

[B23] Osrin D, Tumbahangphe KM, Shrestha D, Mesko N, Shrestha BP, Manandhar MK, Standing H, Manandhar DS, Costello AM Cross sectional, community based study of care of newborn infants in Nepal. BMJ.

[B24] Bolam A, Manandhar DS, Shrestha P, Ellis M, Malla K, Costello AM (1998). Factors affecting home delivery in the Kathmandu Valley, Nepal. Health Policy Plan.

[B25] Sreeramareddy CT, Joshi HS, Sreekumaran BV, Giri S, Chuni N (2006). Home delivery and newborn care practices among urban women in western Nepal: a questionnaire survey. BMC Pregnancy Childbirth.

[B26] Jelliffe DB (1966). The assessment of the nutritional status of the community (with special reference to field surveys in developing regions of the world).

[B27] World Health Organization (1994). Multi-centre study on low birth weight and infant mortality in India, Nepal and Sri Lanka.

[B28] Nepal Demographic and Health Survey (2001). Infant Feeding and Children's and Women's Nutritional Status.

[B29] Central Bureau of Statistics (2002). Population of Nepal, Population Census 2001-Selected Tables. Kathmandu.

[B30] Mullany LC, Darmstadt GL, Khatry SK, Leclerq SC, Tielsch JM (2007). Relationship between the surrogate anthropometric measures, foot length and chest circumference and birth weight among newborns of Sarlahi, Nepal. Eur J Clin Nutr.

[B31] Macdonald PD, Ross SR, Grant L, Young D (2003). Neonatal weight loss in breast and formula fed infants. Arch Dis Child Fetal Neonatal Ed.

[B32] Wright CM, Parkinson KN (2004). Postnatal weight loss in term infants: what is normal and do growth charts allow for it?. Arch Dis Child Fetal Neonatal Ed.

